# Development of the Scale for Measuring Perceived Daily Life Disruption

**DOI:** 10.3390/healthcare11060874

**Published:** 2023-03-16

**Authors:** Jiri Remr

**Affiliations:** INESAN (Institute for Evaluations and Social Analyses), Sokolovská 351/25, 18600 Prague, Czech Republic; jiri.remr@inesan.eu

**Keywords:** disruption, confirmatory factor analysis, COVID-19, validation, psychometrics, coping

## Abstract

The measures that were taken in many countries as a response to the COVID-19 pandemic not only brought about new stimuli in the lives of individuals, but also gave rise to various restrictions that led to discontinuities in many aspects of daily life. Therefore, the development of a sound measure for monitoring the level of perceived daily life disruption is important. The Perceived Daily Life Disruption (PDLD) scale is proposed and its initial validation based on the assessment of key psychometric properties is targeted in this study. A confirmatory factor analysis (CFA) on the PDLD scale was conducted on a representative sample (n = 1372). In addition, univariate statistics were calculated, internal consistency was tested, and unidimensionality based on principal component analysis was performed. The CFA yielded satisfactory results concerning the standard indices and achieved a high internal consistency. It was observed that the scale adequately differentiates the level of perceived daily life disruption among diverse subpopulations. Usage of the PDLD scale can be recommended; it is easy to administer and it yields acceptable results.

## 1. Introduction

A number of anti-epidemic measures have been implemented since March 2020, when the first COVID-19 patients appeared in Czechia and many other countries [1]. The most widespread interventions have been aimed at reducing social interactions; these came in the form of curfews, quarantines, and assembly bans. In order to protect the population from disease transmission, the wearing of facemasks or respirators has been required. At the same time, several other measures have been developed by employers allowed to continue their activity. In this respect, team exchanges, distance (on-line) meetings, and thorough sanitation of the work environment have been introduced and regularly performed.

All these measures have been accompanied by an almost continuous flow of information aimed at (a) justification of the interventions, (b) explanation of their relevance and necessity, (c) increasing attention of individuals with respect to the disease, and (d) behavioral change. Since spring 2020, the topic of contagion, its spread, and its impact on society has dominated the media [2].

Many studies have already analyzed the negative side-effects of the implemented measures on the increased prevalence of mental disorders within the population and specifically within vulnerable sub-populations such as females [3], seniors [4], health-care workers [5], or students [6]. The disease and implemented interventions have brought about a significant change in the lives of most of the population [7]. There are hardly any recognizable sub-groups of the population that remained unaffected. COVID-19 thus represents a significant disruptor of everyday life and the perception of this deserves significant research attention.

The key research questions deal with how individuals reflect on this change, to what extent they perceive such a change as disruptive, and how difficult it is to cope with this new reality.

Attention has recently turned to the perceived disruption and it is usually measured by a single-item indicator. Studies differ in how perceived disruption is defined and what actual variable is used for its indication. For instance, Knepple Carney used the item “The current situation with COVID-19 has been disruptive to your life.” [8] as a component of their broader study focused at the analysis of impacts of COVID-19 as a representant of a large-scale event on well-being of adult population. Similarly, Lee asked the question “How much did your daily life differ because of the COVID-19 outbreak?” within the context of their research that was focused on precautionary behavioral patterns among COVID-19 confirmed patients and quarantined persons [9]. Some other attempts are focused on feelings about the change, merit of stressful situations, or declared severity of impacts. Beside the above-mentioned single-item indicators the more robust measures were developed e.g., Life Events Checklist [10], Impact of Event Scale [11], or Coronavirus Impact Scale [12]. 

My attempt was to develop a reliable, valid, and stable measure that would (a) overcome the issue of a potential response bias associated with single-item measures, (b) minimize the risk of incoherence, and (c) make it possible to compare the perceived disruption in different contexts or among different studies.

In addition to the abovementioned, there is a high demand from public policy makers to identify when the epidemic situation and imposed interventions become unbearable for most of the population [4]. For such a purpose, the Perceived Daily Life Disruption (PDLD) scale was developed and the first attempt to validate it was made. The scale is an instrument that could identify the level of perceived disruption caused by the epidemic situation and corresponding interventions. The scale could be useful for policy-makers to whom it can provide robust feedback informing about changed moods and perceptions of the population. It can also satisfy the needs of public policy analysts to whom it can provide valid dependent variable for their analyses.

The primary aim of this paper is to offer the PDLD scale and provide evidence of its reliability and validity. In this respect, internal consistency was tested, the unidimensional structure of the scale was confirmed, and its psychometric characteristics were analyzed. Moreover, this paper documents the concurrent validity of the PDLD scale by comparing its results with other relevant indicators.

Furthermore, the aim of this paper is to analyze the level (extent) of perceived disruption within some sub-segments of the population. In other words, the mean difference in scores produced by the PDLD scale is analyzed as a function of hypothesized demographic characteristics. For this purpose, the research based on a robust representative sample derived from the general adult population was conducted. Although it was neither necessary for the initial development of the scale nor for its validation, such a research design was useful, as it made it possible to use inferential statistics and compare different subsegments of the total population [13,14].

## 2. Materials and Methods

### 2.1. Research Instrument

PDLD is a five-item scale that might be administered either by participants themselves in e.g., CAWI surveys (Computer Assisted Web Interviewing) or by the interviewers in face-to-face interviews, CAPI (Computer Assisted Personal Interviewing), or CATI surveys (Computer Assisted Telephone Interviewing). Respondents are asked to which extent the statements reflect their attitudes and feelings during the epidemic situation; for this purpose, they were asked to use a five-point Likert-type scale where 0 = very untrue to me, 1 = somewhat untrue to me, 2 = neutral, 3 = somewhat true to me, and 4 = very true to me. The total score ranges from 0 to 20, and a higher score indicates higher perceived disruption.

### 2.2. Scale Development

Initially, 32 statements were extracted from literature, blogs, newspaper articles and other resources. All statements expressed different reflections of the changes in everyday life that the epidemic situation caused. The collection of these 32 statements involved disturbances that individuals might suffer in their everyday lives, discontinuity in daily routines, inability of an individual to perform as usual, increased stress and fears. Five items were excluded from further processing because of overlaps with other statements. Then, the remaining 27 statements were reviewed by an expert panel comprising a sociologist, a psychologist, and a linguist. Based on their evaluation, eight out of the 27 statements were retained (i.e., the other 19 were excluded) and their wording was refined with respect to the purpose of the scale and its theoretical background. After that, the relevance, reasonability, specificity, and understandability of those eight statements were tested on a sample of 23 individuals recruited from the target population. Finally, three items out of the eight statements were excluded due to low understandability and low perceived specificity. At the end, five statements were used for the proposed scale; these statements are listed below.

The overarching idea of the newly developed scale was to inherently involve the dynamics of the change. Other measures, such as perceived quality of life or well-being, are static measures that provide a current snapshot without considering the long-term perspective. They convey current feelings, attitudes, and evaluations. On the other hand, the PDLD scale is designed to mirror the process of change that results in a substantial disruption to everyday life. Refinement of the items was driven by an attempt to differentiate the extent (level) of the perceived disruption. Therefore, the items not only represent a mere inconvenience caused by the change, but also the annoyance and upset; conscious perceived disruption then bridges the reflection of the change with the capacity to cope with changing routines.

### 2.3. Participants and Procedure

The target population is the general population of Czechia aged 15–74 comprising residents permanently living in Czechia.

The applied sampling technique was the multistage random procedure using address-based sampling. Since no adequate sampling frame (register or list of residents) was available, primary sampling units were selected. Within each primary sampling unit, addresses were identified and the appropriate number of households was selected. Finally, field-workers (interviewers) visited pre-selected addresses and attempted to identify prospective respondents using the Kish table [15]. Altogether, 174 primary sampling units throughout Czechia were selected; within each of these units, a maximum of 20 addresses were identified. Interviewers contacted 2657 households and performed 1423 interviews; the response-rate is 53.2 percent. Informed consent had been obtained from each participant before the given interview started. All responses are presented in an aggregated form that makes it impossible to identify a specific person directly or indirectly.

Fieldwork took place during June 2020 (directly after the “first wave” of the COVID-19 outbreak), and the average duration of an interview was approx. 20 min. From all 1423 interviews, 35 percent were supervised by check-backs and verified in terms of compliance with ethical and quality standards. Due to the incompleteness of some interviews, during which respondents refused to provide key socio-demographic data, and due to insincerity of some responses, the datafile comprised 1372 cases used for the analyses. The sample size is large enough to perform scale validation [14,16]. Table 1 shows the structure of the sample with respect to gender, age, and highest achieved education.

Data collection was carried out in the form of face-to-face interviews; field-work was performed after the lockdown when no specific restrictions on social interactions were in place.

### 2.4. Data Analysis

In order to inform about the sample and features of the respondents in the study, the descriptive statistics were calculated, including mean (M), standard deviation (SD) skewness, and kurtosis. Pearsons’s correlation analyses and analyses of variance (ANOVA) were applied to explore and test the differences among relevant measures and indicators. Internal consistency of the scale was tested with the use of Cronbach’s alpha [17,18]. The scale validation comprised the use of exploratory factor analysis with principal components estimation, followed by confirmatory factor analysis (CFA) that was performed in AMOS 24 using the maximum likelihood estimation method, all other analyses were conducted in IBM SPSS ver. 27 (IBM Corp., Armonk, NY, USA).

## 3. Results

### 3.1. Direct Measures

In the given study, self-reported experience of disruption was measured using a total of five indicators. In this respect, the changes as the plausible predictors of perceived disruption focused on physical activities (represented by evaluation of involuntary downturn of the activities that respondents had been enjoying and sufficient number of activities), social life (indicated by the amount of contacts with other people), and selected psychological effects like sleeping disorders (sleep restlessness) and reported amount of stress. 

Moreover, two direct attitudinal questions were posed in order to describe the individuals’ reactions to the pandemic situation. The first direct questions asked about the extent to which respondents were concerned that they might become infected. The second question then asked about the degree of concern about loved ones (i.e., family members or best-friends) becoming infected. These two questions were based on a ten-point scale of concern, where 10 means the respondent is very worried and 1 means not worried at all. The two direct questions regarding the aforementioned concerns were intentionally used as part of the validation strategy. The reason is that the deterioration of one’s own health status as well as the imminent threat to loved ones represent strong disruptors that prevent people from going about their daily lives. One’s own illness, the suddenly appearing need to care for another member of the family, or even the loss of a loved one are not the only significant life-stressors, but they also bring about substantial change and discontinuity. Therefore, my hypothesis was that PDLD scores should increase along with an increase in concerns about oneself and others. The mean score of the concerns about oneself reached 5.26, with a standard deviation of 2.816; the indicator of concerns about others is even higher—its value is 5.61 with a standard deviation of 2.826. 

### 3.2. Quality Criteria

This study is based on classical test theory (CTT), and therefore the scale validation is based on internal consistency, confirmatory factor analysis (CFA), and construct and concurrent validities.

#### 3.2.1. Univariate Statistics

Table 2 shows individual items in the scale followed by key descriptive statistics (i.e., number of valid responses, mean, standard deviation, skewness, kurtosis, and item-total correlation). Relevant statistics are also presented for the given scale as such.

In Table 2, it is evident that the listwise number of responses is 1315 (when analysis of the missing cases does not show any systematic bias); the grand mean is 9.27 (the minimum of this scale is 0 and the maximum is 20), whereas the standard deviation is 4.448. It is also obvious that all standard deviations for the individual items are similar and none produces any specific pattern. The scale produces the following distribution statistics: skewness = −0.186 and kurtosis = −0.521. Shapiro-Wilk test has a value of 0.982 and the Kolmogorov-Smirnov test has a value of 0.086. The item-total correlations range from 0.537 to 0.712.

#### 3.2.2. Uni-Dimensionality and Internal Consistency

The uni-dimensionality of the scale is demonstrated by the results of an exploratory factor analysis. Assuming the eigenvalues are >1, the principal component analysis extracted only one factor that explains 60.7% of the total variance. The Kaiser measure of sampling adequacy is 0.835 and Bartlett’s Test of Sphericity has the following parameters: χ^2^ = 2446.345, df = 10, *p* < 0.001. Factor scores represent the extent to which the item contributes to explaining the whole factor. The higher the value, the more the item contributes to the explanation of the whole factor. All factor loadings are higher than 0.7 when they are ranging from 0.720 (“I struggle to cope with changing routines.”) to 0.837 (“My usual activities are disrupted”).

The value of Cronbach’s alpha is 0.837, and therefore it seems that the scale is inherently stable. Removing any particular item would not lead to a significant improvement in the scale’s consistency.

#### 3.2.3. Psychometric Performance of the Scale

**Figure 1:**
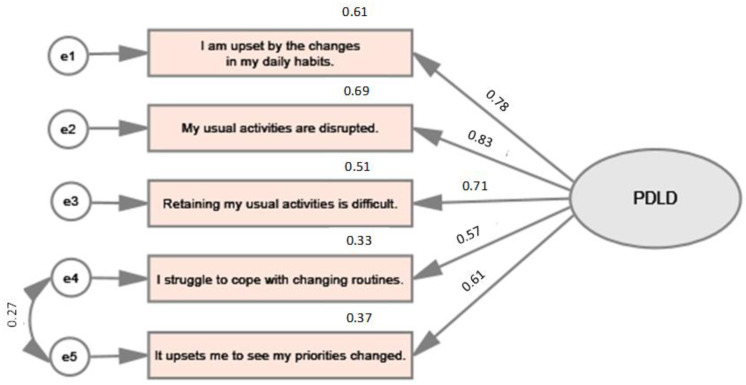
CFA model.

CFA, which tested the hypotheses concerning the extent to which the set of variables represent the expected construct, shows acceptable construct validity. The tested model is depicted in Figure 1.

To assess the model fit, the whole range of indices were calculated. The absolute fit indices comprised χ^2^ = 6.061, df = 4, *p* < 0.195. SRMR (Standardized Root Mean Square Residual) reached 0.0081. Also, RMSEA (Root Mean Square Error of Approximation) was calculated, and its value reached 0.020. In the case of the PDLD scale, the variable-to-factor (VTF) ratio is 5:1. Moreover, the incremental fit indices show a good fit of the model. In this respect, the CFI (Comparative Fit Index) is 0.999 and TLI (Tucker-Lewis Index) is 0.998. 

There are also good reasons to assume convergent validity, which might be understood as the extent to which the items indicate a single underlying construct [19,20,21]. In case of the proposed PDLD scale, the convergent and discriminant validity were identified through AVE (i.e., average variance extracted) and CR (i.e., composite reliability) values when AVE is 0.61 and CR is 0.88.

Concurrent validity is supported by the significant correlation between higher concerns about oneself and higher concerns about loved ones. Pearson’s coefficient is 0.350 (*p* < 0.001) in the case of concerns about oneself and 0.353 (*p* < 0.001) in the case of concerns about others. The scale also produces significant results with other relevant measures reflecting the attitudes and experiences of respondents with COVID-19. For instance, Pearson’s correlation between the PDLD score and concerns about changes in a person’s health is 0.315 (*p* < 0.001). Similarly, the value of correlation between satisfaction and the way individuals spend their time is also significant (r = −0.180; *p* < 0.001); here, the satisfaction scale has a reversed order (1 = very dissatisfied; 5 = very satisfied) and therefore the correlation is negative. 

### 3.3. Comparing the Scores of the PDLD Scale among Subpopulations

Comparability across different subgroups of the general population has also been reviewed. In this respect, Table 3 clearly shows that the scale tends to be stable in terms of key sociodemographic characteristics, when the highest range does not exceed 5% of the continuum [22]. Albeit older respondents, females, and lower-educated respondents show a higher level of disruption, none of those differences is statistically significant (F = 1.784, df = 2, *p* > 0.05 for age; *t*-test = −1.721, df 1313, *p* > 0.05 for gender; F = 1.142, df = 3, *p* > 0.05 for highest achieved education). 

### 3.4. Convergent and Divergent Validity

The PDLD scale sensitively reflects the different experiences [23] that were directly reported by respondents in terms of the change in their everyday routines during the COVID-19 outbreak. ANOVA was used to test the significance of such differences, and its results are shown in Table 4. Overall results are documenting that all between-group differences are significant.

Moreover, convergent validity is supported by the significant positive correlation with higher concerns about self and with higher concerns about closed persons. Pearson’s coefficient is 0.350 (*p* < 0.001) in case of concerns about self, and 0.353 (*p* < 0.001) in case of concerns about the others.

Finally, the divergent validity is supported by the significant negative associations between the PDLD score and reported level of activity during the day. These results, indicated by the agreement with statement “I had a lot of things to do.”, are also presented in Table 4.

## 4. Discussion

The aim of this paper was to evaluate the key psychometric characteristics of the PDLD scale. To attain such a goal, several methods and indices were used, especially internal consistency, principal component analysis, and confirmatory factor analysis. Data show that the PDLD scale performs satisfactorily; results indicate that the scale has acceptable results in terms of distribution when skewness and kurtosis are within the acceptable range of −1.5 to +1.5 [24,25]. As for the item-total correlations, it is acceptable when they are over 0.4 [26]; in this case, such a recommendation is fulfilled when item-total correlations range from 0.537 to 0.712. It seems that the scale also has a uni-dimensional structure, high internal consistency, acceptable construct validity, and good concurrent validity.

The two measures describing the individuals’ reactions to the pandemic situation (i.e., the extent to which respondents were concerned that they might become infected and the degree of concern about loved ones) are considered relevant and reliable in terms of perceived disruption; their significance is reported in many studies [27] or [28].

The achieved value of SRMR (0.0081) is considered acceptable when e.g., Hu and Bentler [29] recommend the SRMR value not to be higher than 0.080. Similarly, RMSEA which is higher than 0.070 is considered by to be an acceptable threshold [30]; in this study its value reached 0.020. Although some authors, e.g., [31] consider VTF ratio of 3:1 to be sufficient, others might perceive five variables per factor as low. With respects to CFI (Comparative Fit Index) and TLI (Tucker-Lewis Index), Hu and Bentler [29] recommend that both indices should exceed 0.95 (in this study CFI is 0.999 and TLI is 0.998. Convergent validity is indicated by average variance extracted (AVE), which should be higher than 0.5 [13], and by composite reliability (CR), which should be higher than 0.6 [14]. To sum up, the PDLD scale shows very good concurrent validity [32] when it is significantly associated with all indicators used in the study [33]. The results thus suggest that the proposed model of the PDLD scale fits in an acceptable way with the data, and the psychometric performance of the scale is supported by the key indices.

The proposed scale seems to be invariant when the differences among key subgroups are not statistically significant; not only the mean scores are very close, but also the standard deviations show similar pattern across the observed subgroups defined by age, gender, and highest achieved education. With respect to other findings e.g., [34,35,36], it would be interesting to use the proposed scale among the selected vulnerable groups and compare the differences obtained.

The scale that is focused on perceived daily life disruption is a novel one among COVID-19 measures (these are typically focused on fear, stress, anxiety, etc.). However, perceived daily life disruption also appears within other contexts, such as reactions to natural disasters [37], distress and hardship in family bonds, adolescent transition [38], and other rapid and complex changes to daily routines [39]. 

Since the conducted research took the form of a cross-sectional study, it is not possible to identify the direction of association between perceived daily life disruption and perceived amount of stress and reported sleeping disorders. It is not possible to recognize whether increased amount of stress and reported sleeping disorders lead to a higher perceived disruption or whether a higher perceived disruption concerning COVID-19 leads to increased amount of stress and reported sleeping disorders. Both explanations would have their own rationale; however, another type of study would be required to determine the direction of causality.

There was a specific situation in Czechia during fieldwork. The interviews were conducted right after the so-called “first wave” of the pandemic, which may be characterized by the imposing of measures on the population (a lockdown lasting for weeks, among others), a high level of solidarity among individuals (mutual help), and good compliance with restrictions. It would be useful to compare the results with newer data to observe the maturation effect in perceived daily life disruption.

## 5. Conclusions

The paper provides evidence that expands the growing body of research on the effects of the pandemic on the population. The study has shed some light on plausible predictors, moderators, and mediators of perceived daily life disruption. The scale is easy to deploy and administer when it comprises only five statements. Items are not sensitive for a major part of the population and they do not impose a large burden on either interviewers or respondents.

The PDLD scale provides useful feedback concerning perception of this aspect of the epidemic situation. It might serve as an indicator for evaluating interventions, monitoring the impact of COVID-19 on the population over time, and comparing different target groups. The large sample size helps to increase the generalizability of the research estimates and improve the accuracy of achieved outcomes. The added value of the PDLD scale is two-fold: the scale (a) provides more stable evidence than the direct question and (b) it might serve as a dependent variable of a higher level of measurement compared to currently used binary or ordinal variables.

The newly developed scale was proved by the presented study to be reliable, internally consistent, and valid instrument to measure the level of perceived disruption of daily activities caused by COVID-19 outbreak. The study showed good properties of the scale; PDLD is invariant among subpopulations defined by gender, age, or education, and is sensitive to such life-disruptors as it was the COVID-19 outbreak.

Even though the PDLD scale was developed in the context of the COVID-19 outbreak, it is a versatile tool that can help to understand how individuals manage other types of life stressors. As such, it might be used for different kinds of societal changes, not only for the COVID-19 situation. The suitability and performance of the proposed scale should indeed be tested in a wide variety of contexts. Future research could examine the correlations between the PDLD scale and anxiety and depressive symptoms (e.g., PHQ-9), fear (e.g., FCV-19S), anxiety (GAD-7), but also well-being, perceived quality of life, or coping strategies.

## Figures and Tables

**Table 1:** Selected socio-demographic characteristics of the sample.

Variables		
Gender	Male	50.0%
	Female	50.0%
	Total	100.0%
Age	15–29 years	19.8%
	30–49 years	40.0%
	50–74 years	40.2%
	Total	100.0%
Highest achieved education	Elementary	7.7%
	Vocational	35.4%
	Secondary	42.4%
	University	14.5%
	Total	100.0%

Note: n = 1372.

**Table 2:** PDLD scale—descriptive statistics.

Items		N	Mean	SD	Skewness	Kurtosis	Item-Total Correlation
1	My usual activities are disrupted.	1315	1.90	1.189	−0.048	−0.821	0.712
2	It upsets me to see my priorities changed.	1315	1.66	1.151	0.107	−0.813	0.606
3	I struggle to cope with changing routines.	1315	1.62	1.139	0.157	−0.789	0.573
4	I am upset by the changes in my daily habits.	1315	2.14	1.145	0.243	−0.611	0.683
5	Retaining my usual activities is difficult.	1315	1.95	1.090	−0.139	−0.633	0.622
PDLD Scale (the whole scale)		1315	9.27	4.448	−0.186	−0.521	

**Table 3:** PDLD scores within the selected subpopulations.

		PDLD Score M (SD)
Age	15–29 years (19.8%)	8.94 (4.60)
	30–49 years (40.0%)	9.16 (4.48)
	50–74 years (40.2%)	9.53 (4.33)
Gender	Male (50.0%)	9.06 (4.52)
	Female (50.0%)	9.48 (4.37)
Highest achieved education	Elementary (7.7%)	9.61 (4.04)
	Vocational (35.4%)	9.58 (4.11)
	Secondary (42.4%)	9.15 (4.33)
	University (14.5%)	8.64 (4.63)
	Total	9.27

**Table 4:** PDLD scores by experienced disruption.

		M (SD)	
Involuntary downturn of the activities I enjoy.	substantial (15.6%)	11.11 (3.84)	F = 53.231, df = 2, *p* < 0.001
	partial (30.8%)	10.26 (3.85)	
	minor (53.6%)	8.16 (4.62)	
Reduction of social contacts.	substantial (34.5%)	11.8 (3.95)	F = 200.227, df = 2, *p* < 0.001
	partial (34.0%)	9.4 (3.62)	
	minor (31.5%)	6.4 (4.13)	
Increased sleep restlessness.	substantial (20.2%)	12.4 (3.60)	F = 216.637, df = 2, *p* < 0.001
	partial (30.0%)	10.7 (2.98)	
	minor (49.8%)	7.1 (4.38)	
Increased amount of stress.	substantial (25.7%)	12.4 (3.35)	F = 249.025, df = 2, *p* < 0.001
	partial (30.2%)	10.2 (3.37)	
	minor (44.1%)	6.8 (4.28)	
“I had a lot of things to do.”	agree (53.3%)	8.28 (4.66)	F = 45.368, df = 2, *p* < 0.001
	neither, nor (30.9%)	9.96 (4.05)	
	disagree (15.8%)	11.24 (3.51)	
	Total	9.27	

## Data Availability

The data used to support the findings of this study will be available from the corresponding author upon reasonable request.
